# Persistence of Infarct Zone T2 Hyperintensity at 6 Months After Acute ST-Segment–Elevation Myocardial Infarction: Incidence, Pathophysiology, and Prognostic Implications

**DOI:** 10.1161/CIRCIMAGING.117.006586

**Published:** 2017-12-14

**Authors:** Jaclyn Carberry, David Carrick, Caroline Haig, Nadeem Ahmed, Ify Mordi, Margaret McEntegart, Mark C. Petrie, Hany Eteiba, Stuart Hood, Stuart Watkins, Mitchell Lindsay, Andrew Davie, Ahmed Mahrous, Ian Ford, Naveed Sattar, Paul Welsh, Aleksandra Radjenovic, Keith G. Oldroyd, Colin Berry

**Affiliations:** From the BHF Glasgow Cardiovascular Research Centre, Institute of Cardiovascular and Medical Sciences (J.C., D.C., N.A., I.M., M.M., M.C.P., H.E., S.H., S.W., M.L., A.D., A.M., N.S., P.W., A.R., K.G.O., C.B.) and Robertson Centre for Biostatistics (C.H., I.F.), University of Glasgow, Scotland; and West of Scotland Heart and Lung Centre, Golden Jubilee National Hospital, Clydebank (D.C., S.W., C.B.).

**Keywords:** acute coronary syndrome, magnetic resonance imaging, myocardial infarction, myocardium, prognosis

## Abstract

**Background—:**

The incidence and clinical significance of persistent T2 hyperintensity after acute ST-segment–elevation myocardial infarction (STEMI) is uncertain.

**Methods and Results—:**

Patients who sustained an acute STEMI were enrolled in a cohort study (BHF MR-MI: NCT02072850). Two hundred eighty-three STEMI patients (mean age, 59±12 years; 75% male) had cardiac magnetic resonance with T2 mapping performed at 2 days and 6 months post-STEMI. Persisting T2 hyperintensity was defined as infarct T2 >2 SDs from remote T2 at 6 months. Infarct zone T2 was higher than remote zone T2 at 2 days (66.3±6.1 versus 49.7±2.1 ms; *P*<0.001) and 6 months (56.8±4.5 versus 49.7±2.3 ms; *P*<0.001). Remote zone T2 did not change over time (mean change, 0.0±2.7 ms; *P*=0.837), whereas infarct zone T2 decreased (−9.5±6.4 ms; *P*<0.001). At 6 months, T2 hyperintensity persisted in 189 (67%) patients, who were more likely to have Thrombus in Myocardial Infarction flow 0 or 1 in the culprit artery (*P*=0.020), incomplete ST-segment resolution (*P*=0.037), and higher troponin (*P*=0.024). Persistent T2 hyperintensity was associated with NT-proBNP (N-terminal pro-B-type natriuretic peptide) concentration (0.57 on a log scale [0.42–0.72]; *P*=0.004) and the likelihood of adverse left ventricular remodeling (>20% change in left ventricular end-diastolic volume; 21.91 [2.75–174.29]; *P*=0.004). Persistent T2 hyperintensity was associated with all-cause death and heart failure, but the result was not significant (*P*=0.051). ΔT2 was associated with all-cause death and heart failure (*P*=0.004) and major adverse cardiac events (*P*=0.013).

**Conclusions—:**

Persistent T2 hyperintensity occurs in two thirds of STEMI patients. Persistent T2 hyperintensity was associated with the initial STEMI severity, adverse remodeling, and long-term health outcome.

**Clinical Trial Registration—:**

URL: http://www.clinicaltrials.gov. Unique identifier: NCT02072850.

## Abstract 2

Supplemental Digital Content is available in the text.

In survivors of acute ST-segment–elevation myocardial infarction (STEMI), edema within the infarct zone revealed by T2-weighted cardiac magnetic resonance (CMR) imaging^1,2^ is associated with the initial extent of myocardial jeopardy,^3^ the size of infarction,^4^ and prognosis in the longer term.^5^ Edema impairs myocardial contractility by reducing the binding efficiency of actin–myosin filaments leading to reduced force generation in affected cardiomyocytes.^6^

**See Editorial by Dharmakumar**

**See Clinical Perspective**

There is uncertainty about the natural history and clinical significance of persistently high infarct zone T2 values because previous studies were limited by sample size (n=10–62),^7–10^ or method of detection,^7,8^ for example, T2-weighted short inversion time inversion recovery (STIR) imaging. Contemporary quantitative T2-mapping techniques have better diagnostic accuracy^11^ and repeatability^12^ compared with T2-weighted STIR imaging.

Our aims were to (1) measure infarct zone T2 (ms) and its changes over time in a longitudinal study of acute STEMI patients; (2) determine the incidence of persistent T2 hyperintensity at 6 months post-STEMI; (3) assess the clinical characteristics and left ventricular (LV) size and function of those patients with persistent T2 hyperintensity and compare them to those patients in whom T2 hyperintensity had resolved; and (4) assess the association of persisting T2 hyperintensity with longer-term health outcome.

We hypothesized that the persistence of myocardial infarct zone T2 hyperintensity would be associated with the initial STEMI severity, and it would be associated with surrogate measures of outcome, including LV volume and NT-proBNP (N-terminal pro-B-type natriuretic peptide), and longer-term health outcome.

## Methods

The full methodology has been reported previously (BHF MR-MI [Detection and Significance of Heart Injury in ST Elevation Myocardial Infarction]: NCT02072850) and is detailed in the Methods in the Data Supplement. Patients with acute STEMI were consecutively screened for suitability and those recruited provided written informed consent. The study was approved by the National Research Ethics Service (Reference 10-S0703-28) and was publically registered (NCT02072850). The data, analytic methods, and study materials will be made available to other researchers for purposes of reproducing the results or replicating the procedure.^13^

### CMR Image Analyses

#### Myocardial Edema

CMR images were analyzed on a Siemens workstation. The epicardial and endocardial contours on the last corresponding T2-weighted raw image with an echo time of 55 ms were planimetered and copied to the T2 map.^14^ Regions of interest were drawn in the remote and infarct zones to measure the respective signal intensities. T2 hyperintensity was present if the T2 signal in the infarct zone was 2 SDs above the T2 signal in the remote zone.^11,15^ Areas of microvascular obstruction or hemorrhage, identified by consulting late gadolinium enhancement and T2* images, respectively, were excluded from the infarct region of interest because this would reduce the signal intensity and may mask the presence of T2 hyperintensity. The remote zone was drawn 180° from infarcted myocardium, midmyocardial, and ≈1 segment in length. Measurement was performed on multiple slices and the average taken.

#### Infarct Definition and Size

The territory of infarction was delineated using a signal intensity threshold of >5 SD above a remote reference region and expressed as a percentage of total LV mass.^16^

#### Myocardial Salvage

Myocardial salvage was calculated by subtraction of percent infarct size from percent myocardial edema.^5,17,18^ The myocardial salvage index was calculated by dividing the myocardial salvage area by the initial extent of edema.

#### Adverse Remodeling

Adverse remodeling was defined as an increase in LV end-diastolic volume at 6 months from baseline by ≥20%.^19^

### Health Outcomes

We prespecified adverse health outcomes that are pathophysiologically linked with STEMI. The primary composite outcome was major adverse cardiac events (MACE) defined as cardiac death, nonfatal myocardial infarction, or heart failure hospitalization after the 6-month CMR scan. All-cause death or heart failure (heart failure hospitalization or defibrillator implantation) after the 6-month CMR scan was a secondary outcome.

### Statistics

The full statistical methods are reported in the Data Supplement. All *P* values were 2-sided. A *P* value >0.05 indicated the absence of a statistically significant effect. Analyses were performed using SPSS version 22 for Windows (SPSS, Inc, Chicago, IL) or R v3.3.0.

## Results

Of 343 STEMI patients referred for emergency percutaneous coronary intervention, 283 (87%) patients with paired scans were included in the final analyses. The flow diagram for the study is shown in Figure I in the Data Supplement.

### Patient Characteristics

Using T2 mapping, 189 (67%) patients had persistent T2 hyperintensity at 6 months post-STEMI.

Patient characteristics are shown in Table 1. The mean age was 59±11 years, and 75% were male. Patients with persisting T2 hyperintensity were more likely to present with Thrombus in Myocardial Infarction flow 0 or 1 in the culprit artery (Thrombus in Myocardial Infarction flow 0 and 1: 61 [64%] without versus 144 [76%] with persisting T2 hyperintensity; Thrombus in Myocardial Infarction flow 2: 18 [19%] versus 34 [18%]; Thrombus in Myocardial Infarction flow 3: 15 [16%] versus 11 [6%]; *P*=0.020). They were more likely to have partial resolution of the ST-segment postreperfusion (none: 15 [16%] versus 26 [14%]; partial: 25 [27%] versus 79 [42%]; complete: 54 [57%] versus 83 [44%]; *P*=0.037) and had higher troponin levels post-STEMI (1126 [155–3814] versus 2095 [122–5550] ng/L; *P*=0.024). Other clinical characteristics of patients with and without persisting T2 hyperintensity were similar (*P*>0.050).

**Table 1.**
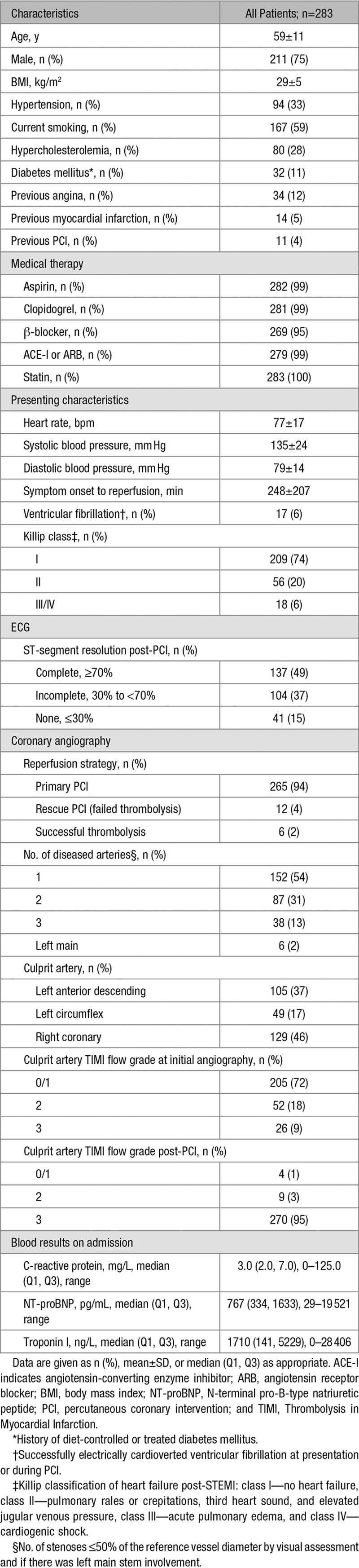
Characteristics of 283 Patients With Acute STEMI

### CMR Findings

#### During the Index Hospitalization

**Figure 1.**
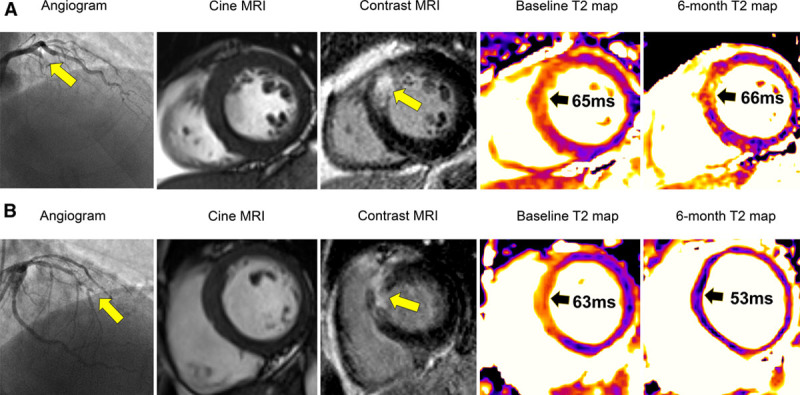
Two patients with a similar presentation of acute anterior ST-segment–elevation myocardial infarction. Both patients were treated by percutaneous coronary intervention and with the same antithrombotic drugs. At the end of the procedure, both patients had Thrombus in Myocardial Infarction (TIMI) coronary flow grade 3 in the culprit left anterior descending artery. **A**, A patient with persistent infarct zone T2 hyperintensity: cardiac magnetic resonance (CMR) imaging was performed 2 days post-revascularization. T2 mapping revealed an infarct zone T2 value of 65 ms. CMR performed at 6 mo revealed a persistently high infarct zone T2 value of 66 ms in a matched myocardial slice position to baseline. Left ventricular (LV) end-diastolic volume increased from 143 to 175 mL at 6 mo representing adverse remodeling. This patient was readmitted with heart failure after the 6-mo CMR scan. **B**, A patient without persistent infarct zone T2 hyperintensity: CMR was performed 2 days post-revascularization. T2 mapping revealed an infarct zone T2 value of 63 ms. CMR performed at 6 mo revealed a lower infarct zone T2 value of 53 ms. LV end-diastolic volume decreased from 120 to 118 mL at 6 mo. This patient had an uncomplicated clinical course. MRI indicates magnetic resonance imaging.

CMR findings are summarized in Table 2 and Table I in the Data Supplement. Exemplar clinical cases are included in Figure 1. At 2 days, the T2 signal in the infarct zone was higher than in the remote zone (66.3±6.1 versus 49.7±2.1 ms; *P*<0.001).

**Table 2.**
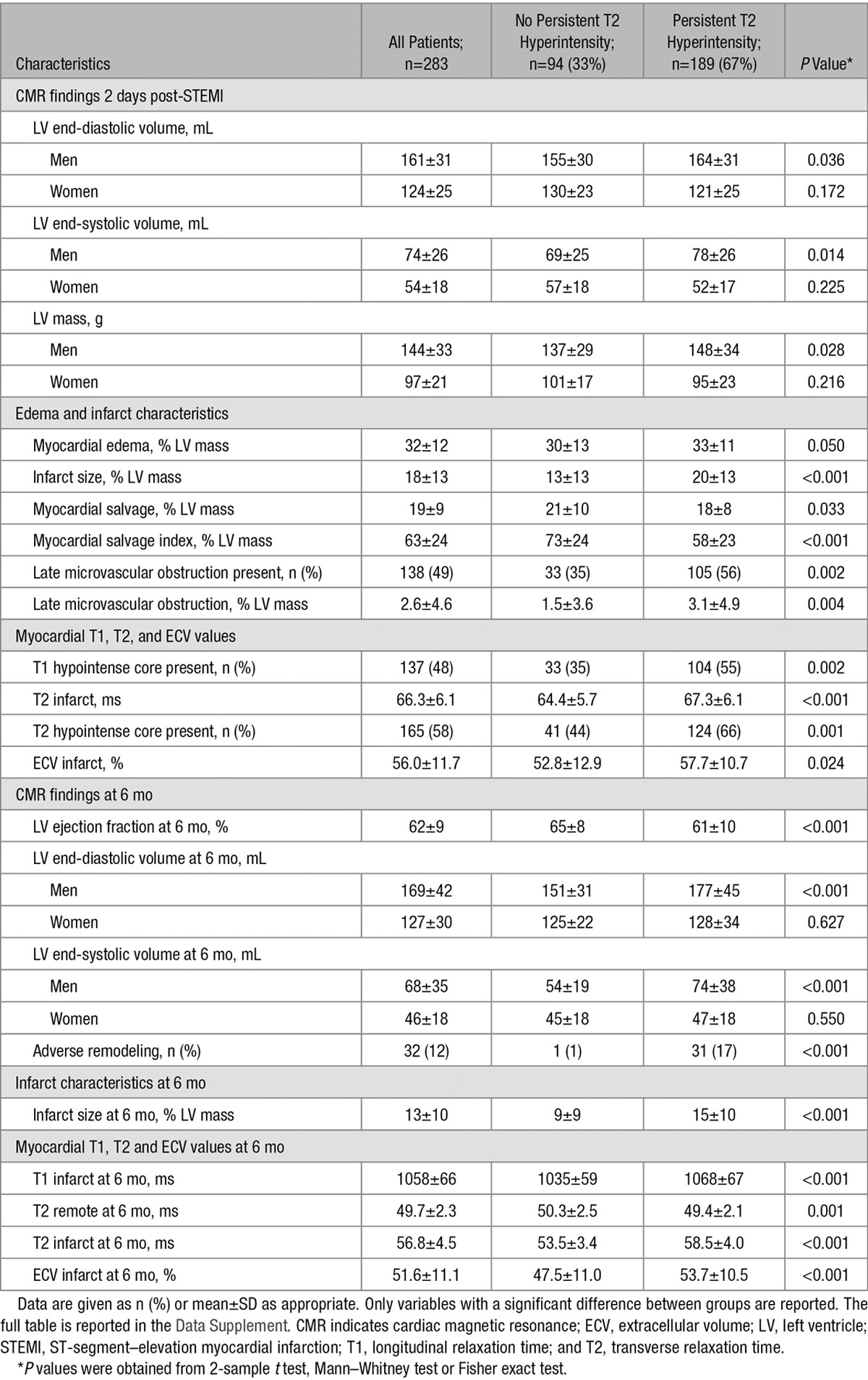
CMR Findings in 283 Patients Grouped According to the Presence or Absence of Persistent T2 Hyperintensity Revealed by T2 Mapping at 6 Months Post-STEMI

Patients with persisting T2 hyperintensity had higher LV volumes, more extensive infarcts, and lower myocardial salvage indexes (Table 2). They were more likely to have microvascular obstruction, a larger extent of microvascular obstruction, and T2 signal and extracellular volume in the infarct zone were significantly higher than in those without persisting T2 hyperintensity (Table 2). There was an association between the extent of microvascular obstruction and the extent of myocardial edema at 2 days (0.21% [0.17%–0.25%]; *P*<0.001) and a trend to association in the extent of myocardial edema at 2 days post-STEMI and the persistence of T2 hyperintensity (Table 2). There was no difference in T1 or T2 core signal between patients with and without persisting T2 hyperintensity (Table I in the Data Supplement).

The results of interobserver agreement of infarct zone T2 measurements are shown in Figure II in the Data Supplement.

#### At 6 Months

**Figure 2.**
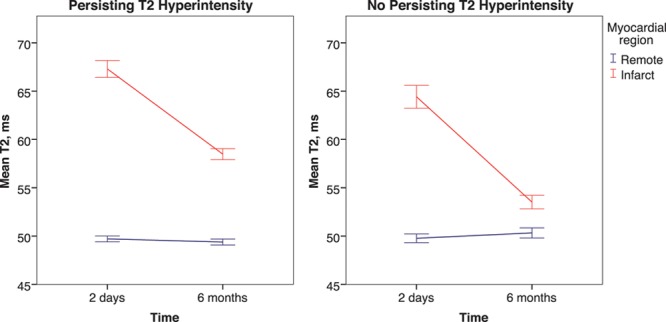
Change in T2 signal in patients with ST-segment–elevation myocardial infarction with or without persisting infarct zone T2 hyperintensity at 6 mo. Infarct zone T2 decreases in the majority of patients but to a lesser degree in patients with persisting edema.

T2 remained higher in the infarct zone compared with the remote zone at 6 months (56.8±4.5 versus 49.7±2.3 ms; *P*<0.001). Remote zone T2 did not change between day 2 and 6 months (mean change, 0.0±2.7 ms; *P*=0.837), whereas infarct zone T2 decreased (−9.5±6.4 ms; *P*<0.001; Figure 2). Patients with persistent T2 hyperintensity had a smaller reduction in infarct zone T2 (−8.8±6.6 versus −10.9±6.0 ms; *P*=0.010). The change in infarct zone T2 was associated with the extent of microvascular obstruction at 2 days (0.20% [0.08%–0.33%]; *P*=0.002).

At 6 months, those with persisting T2 hyperintensity had lower LV ejection fractions and larger LV volumes. Infarct size remained larger in those with persisting T2 hyperintensity, and infarct zone CMR parameters were higher (T1, T2, and extracellular volume; Table 2). Remote zone T2 signal was lower in those with persisting T2 hyperintensity, whereas remote zone T1 signal was the same (Table 2; Table I in the Data Supplement).

**Figure 3.**
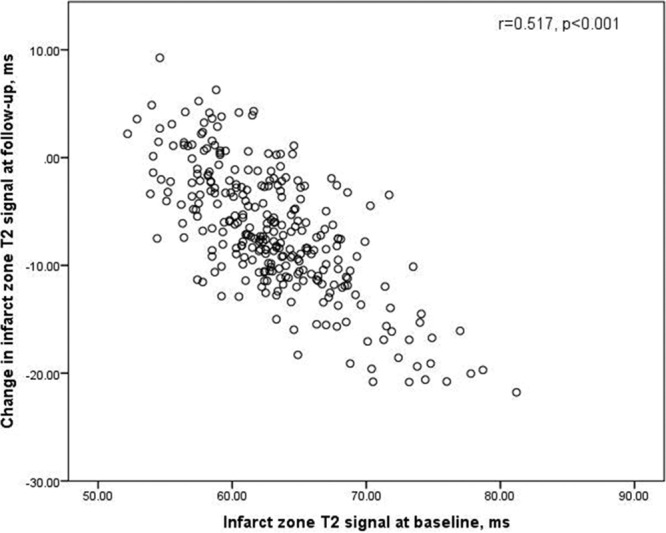
Change in infarct zone T2 vs infarct zone T2 at baseline. Infarct zone T2 at baseline was negatively associated with the change in infarct zone T2 at 6 mo.

The higher the initial infarct zone T2 signal, the larger the decrease in infarct zone T2 signal by 6 months (Figure 3).

### Persistent T2 Hyperintensity and LV Remodeling

Adverse remodeling occurred in 32 (12%) patients (Table 2). In a binary logistic regression analysis, persistent T2 hyperintensity was a multivariable associate of adverse remodeling (Table 3). When the change in infarct zone T2 (1 ms change and 10 ms change) was included in place of persistent T2 hyperintensity at 6 months, this was also associated with adverse remodeling (Table 3). When the change in infarct zone extracellular volume was included in the multivariable models, persistent T2 hyperintensity and change in infarct zone T2 were not associated with the change in LV end-diastolic volume.

**Table 3.**
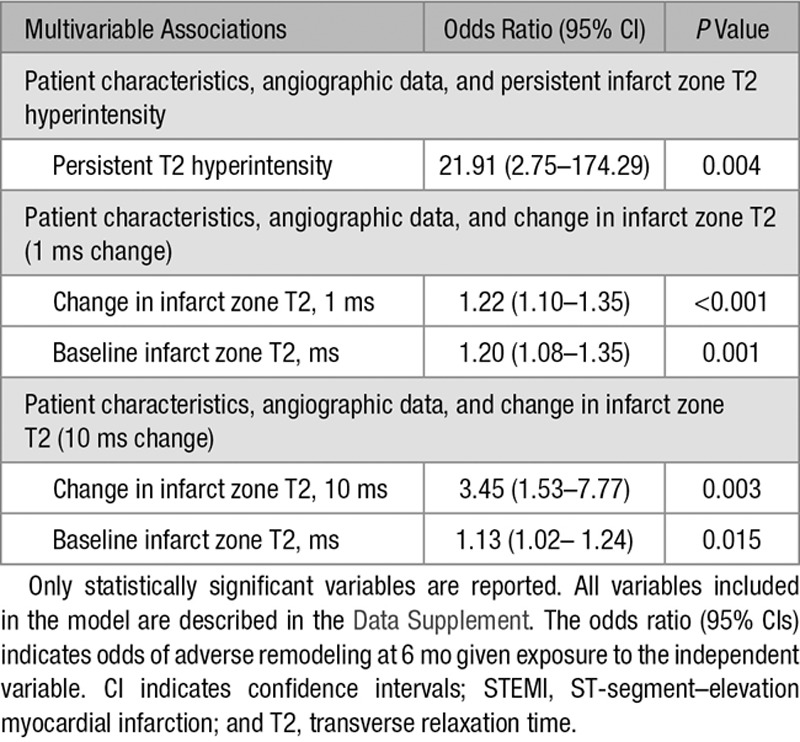
Binary Logistic Regression Analysis for Associations With Adverse Remodeling at 6 Months Post-STEMI in 283 Patients

### Persistent T2 Hyperintensity and LV Function at 6 Months

At 2 days, LV ejection fraction was similar between patients with and without persisting T2 hyperintensity, whereas 6-month ejection fraction was lower in those with persisting T2 hyperintensity (Table 2). The mean change in LV ejection fraction was 6.7±7.8%. Patients with persisting T2 hyperintensity had a numerically lower increase in LV ejection fraction without statistical significance (6.1±7.8% versus 7.9±7.7%).

The change in LV ejection fraction was associated with both persisting T2 hyperintensity and the change in infarct zone T2 (Table II in the Data Supplement).

### Persistent T2 Hyperintensity and NT-proBNP

Blood samples were collected in the participants who were enrolled during office hours (n=123 patients at baseline and n=98 patients at follow-up). The characteristics of these patients were similar to the whole cohort (data not shown). Persistent T2 hyperintensity was associated with NT-proBNP at 6 months (0.57 on a log scale [0.42–0.72]; *P*=0.004), but not at baseline.

### Persistent T2 Hyperintensity and Health Outcomes

Health outcome data were available in 283 (100%) patients. The median duration of follow-up was 1330 days (minimum–maximum postdischarge censor duration 794–1622 days). All-cause death or heart failure occurred in 19 (7%) patients, including 7 noncardiovascular deaths, 4 cardiovascular deaths, 1 stroke death, 1 undetermined cause of death, and 6 heart failure episodes. Sixteen (6%) patients experienced a MACE after the CMR scan at 6 months, including 6 heart failure episodes (Killip class 3 or 4 heart failure or defibrillator implantation), 4 cardiovascular deaths, 4 admissions with non-STEMI, and 2 admissions with STEMI.

Persisting T2 hyperintensity (binary, yes or no) was associated with the occurrence of all-cause death or heart failure (hazard ratio, 4.31; 95% confidence interval, 1.00–18.67; *P*=0.051); however, the association was not statistically significant. Persisting T2 hyperintensity was not associated with MACE (Figure III in the Data Supplement).

The change in infarct zone T2 (1 ms change) was associated with all-cause death or heart failure (hazard ratio, 1.15; 95% confidence interval, 1.05–1.27; *P*=0.004) and with MACE (hazard ratio, 1.14; 95% confidence interval, 1.03, 1.27; *P*=0.013). Similar results were observed when considering a 10 ms change in infarct zone T2 (all-cause death or heart failure hazard ratio, 3.77; 95% confidence interval, 1.58–8.97; *P*=0.003: MACE hazard ratio, 3.60; 95% confidence interval, 1.42–9.15; *P*=0.007).

## Discussion

We present a natural history study of the changes in infarct zone T2 over time and prognostic significance over 4 years in a large unselected cohort of STEMI patients.

The main findings are as follows: (1) T2 hyperintensity persisted in approximately two thirds of patients at 6 months post-STEMI; (2) infarct zone T2 decreased in the long term in most patients, and the decrease was larger in patients with higher infarct zone T2 at baseline; (3) persisting T2 hyperintensity was associated with electrocardiographic, angiographic, CMR, and biochemical markers of STEMI severity including the initial size of infarction, presence of microvascular obstruction, the myocardial salvage index, peak troponin, and NT-proBNP; (4) persistent T2 hyperintensity and the change in infarct zone T2 were associated with adverse remodeling and worsening LV function; and (5) the change in infarct zone T2 was associated with adverse health outcomes. T2 hyperintensity in the infarct zone that persists at 6 months post-STEMI is an adverse prognostic sign and presents a mechanistic explanation for worsening LV volumes and function.

Direct comparison with previous studies^7–10^ is qualified by differences in sample size and imaging methods. These previous studies have defined persisting T2 hyperintensity as edema; however, strong evidence that elevated infarct zone T2 at 6 months post-STEMI represents edema is lacking. Histological studies validating T2 signal as a representation of edema have been focused on the acute phase post-STEMI.^20–22^ Infarct zone edema as a cause of persisting T2 hyperintensity at 6 months cannot be excluded; however, other causes of increased myocardial mobile water content such as myocardial fat should be considered.

The incidence of persistent T2 hyperintensity in the present study was high (two thirds of patients) in comparison to some^9,10^ but not all prior recent studies.^7,8^ In those studies, the numbers of participants with paired data were limited (n=10–62)^7–10^ implying imprecision.

Ripa et al^7^ found that myocardial T2 hyperintensity (defined as edema) persisted in 51 of 54 (94%) STEMI patients using T2 STIR imaging. The average number of affected segments per patient decreased by 4.5 segments at 6 months. Persistent edema was not associated with LV ejection fraction.

Nilsson et al^8^ identified a prevalence of high T2 at 6 months which was comparable with our result (60%); however, this analysis was performed on a small sample, using T2 STIR imaging, and clinical and prognostic information was limited. Dark blood STIR edema imaging is a qualitative technique^11^ with reduced diagnostic accuracy, when compared with quantitative T2 mapping.^12^ The authors speculate that T2 hyperintensity may represent edema or an alternative process, such as hemoglobin breakdown products or increased unbound water.^8^

Dall’Armellina et al^9^ used bright blood T2-weighted CMR (n=23 [77%] with paired data). They found that 35% of myocardial segments had evidence of edema acutely, and the proportion of edematous segments reduced to 6%, with a small number of cases having infarct zone edema at 6 months. They found that a reduction in edema was associated with an improvement in wall motion score index.

Zia et al^10^ used T2 mapping and found that infarct zone T2 signal equalizes with remote zone T2 signal at 6 months suggesting complete recovery of myocardial edema. There are some reasons why the results of this article may be so different to those we present. The sample size was limited (n=62 compared with our n=283).^10^ The methodology refers to infarct segment rather than zone.^10^ If infarct T2 signal has been measured across an entire myocardial segment, then that segment may also contain unaffected myocardium (and hypointense core) and therefore bias the results by averaging the signal across 2 myocardial states.

Our study is the first and largest to use contemporary, quantitative methods to identify the incidence and clinical significance of persistent T2 hyperintensity in a cohort of near-consecutive patients with acute STEMI. Our study presents new insights. The adverse clinical significance of persistent T2 hyperintensity was underscored by its associations with the initial STEMI severity and LV remodeling, and the validity of our observations is enhanced given that the T2 maps were of diagnostic quality in nearly all patients.

We saw that there was a larger extent of acute microvascular obstruction in those with persisting T2 hyperintensity, and there was also an association between the extent of microvascular obstruction and the extent of myocardial edema acutely. The relationship between microvascular obstruction and myocardial edema may provide a mechanistic explanation into the persistence of T2 hyperintensity, specifically because evidence has shown myocardial hemorrhage, which is related to microvascular obstruction, leads to iron driven inflammation.^23,24^ We also observed worsening LV ejection fractions in patients with persisting T2 hyperintensity, which is in keeping with previous reports of edema and attenuated strain.^25^

Previous research from this cohort suggests that there is a significant difference in remote zone T2 between the 2 time points,^26,27^ which may be explained by the larger sample size in the present study (n=30 in Carrick et al^26^ and n=131 in Carberry et al^27^). Persisting T2 hyperintensity may reflect the natural history of infarct healing^3^ and inflammation,^28^ and potentially, latency of edema within the infarct zone to dissipate, especially if water content is substantially increased acutely. Nonetheless, we observed that persistent T2 hyperintensity has adverse prognostic implications based on its association with LV remodeling and health outcome. The reason why the change in infarct zone T2 was associated with MACE and persisting T2 hyperintensity was not explained by the nature of the data. Dichotomizing a continuous variable can reduce statistical power. In addition, our results may simply reflect the fact that the absolute change in T2 signal in the infarct zone is more predictive of MACE than the presence or absence of T2 hyperintensity. Because the analysis was limited by the event rate, further research is warranted.

### Limitations

Because of the length of the imaging protocol, we restricted other imaging methods, such as for myocardial fat. The survival analysis was limited by the absolute number of heart failure and all-cause death events (n=19) and MACE events (n=16), so these results should be interpreted carefully and taken as hypothesis generating.

### Conclusions

Infarct zone T2 hyperintensity persisted at 6 months in approximately two thirds of STEMI patients. Persistent T2 hyperintensity was prognostically important because it was associated with markers of STEMI severity and adverse LV remodeling. The change in infarct zone T2 signal as a continuous variable was associated with health outcome. Whether T2 hyperintensity at 6 months represents edema or another process is uncertain and merits further discussion and study. Further studies are warranted to assess whether or not infarct zone T2 may track the response to therapy in STEMI patients and thus represent a therapeutic target for use in clinical trials.

## Acknowledgments

We thank the patients and the staff in the Cardiology and Radiology Departments. We thank Peter Weale and Patrick Revell (Siemens Healthcare, United Kingdom).

## Sources of Funding

The British Heart Foundation (PG/11/2/28474; RE/13/5/30177) and the Chief Scientist Office of the Scottish Government supported this research. C. Berry was supported by a Senior Fellowship from the Scottish Funding Council. Dr Welsh is supported by BHF Fellowship FS/12/62/29889. This project was supported by a research agreement with Siemens Healthcare.

## Disclosures

None.

## Supplementary Material

## CLINICAL PERSPECTIVE

In survivors of acute ST-segment–elevation myocardial infarction, edema within the infarct zone is associated with the initial extent of myocardial jeopardy, the size of infarction, and prognosis. Edema impairs myocardial contractility by reducing the binding efficiency of actin–myosin filaments leading to reduced force generation in affected cardiomyocytes. There is uncertainty about the natural history and clinical significance of the persistence of T2 hyperintensity because previous studies were limited by sample size or method of detection. We present a natural history study of the change in infarct zone T2 signal over time and prognostic significance over 4 years in a large unselected cohort of ST-segment–elevation myocardial infarction patients and using contemporary, quantitative T2-mapping techniques. Infarct zone T2 hyperintensity was present at 6 months in approximately two thirds of ST-segment–elevation myocardial infarction patients. Persistent T2 hyperintensity was prognostically important because it was associated with markers of MI severity and adverse left ventricular remodeling. Whether T2 hyperintensity at 6 months represents edema or another process is uncertain and merits further discussion and study. The change in infarct zone T2 signal as a continuous variable was associated with health outcome. Further studies are warranted to assess whether or not infarct zone T2 may track the response to therapy in ST-segment–elevation myocardial infarction patients and thus represent a therapeutic target for use in clinical trials.

## References

[1] Kuntz ID, Brassfield TS, Law GD, Purcell GV (1969). Hydration of macromolecules.. Science.

[2] Karolle BL, Carlson RE, Aisen AM, Buda AJ (1991). Transmural distribution of myocardial edema by NMR relaxometry following myocardial ischemia and reperfusion.. Am Heart J.

[3] Berry C, Kellman P, Mancini C, Chen MY, Bandettini WP, Lowrey T, Hsu LY, Aletras AH, Arai AE (2010). Magnetic resonance imaging delineates the ischemic area at risk and myocardial salvage in patients with acute myocardial infarction.. Circ Cardiovasc Imaging.

[4] Kim HW, Van Assche L, Jennings RB, Wince WB, Jensen CJ, Rehwald WG, Wendell DC, Bhatti L, Spatz DM, Parker MA, Jenista ER, Klem I, Crowley AL, Chen EL, Judd RM, Kim RJ (2015). Relationship of T2-weighted MRI myocardial hyperintensity and the ischemic area-at-risk.. Circ Res.

[5] Eitel I, Desch S, Fuernau G, Hildebrand L, Gutberlet M, Schuler G, Thiele H (2010). Prognostic significance and determinants of myocardial salvage assessed by cardiovascular magnetic resonance in acute reperfused myocardial infarction.. J Am Coll Cardiol.

[6] Bragadeesh T, Jayaweera AR, Pascotto M, Micari A, Le DE, Kramer CM, Epstein FH, Kaul S (2008). Post-ischaemic myocardial dysfunction (stunning) results from myofibrillar oedema.. Heart.

[7] Ripa RS, Nilsson JC, Wang Y, Søndergaard L, Jørgensen E, Kastrup J (2007). Short- and long-term changes in myocardial function, morphology, edema, and infarct mass after ST-segment elevation myocardial infarction evaluated by serial magnetic resonance imaging.. Am Heart J.

[8] Nilsson JC, Nielsen G, Groenning BA, Fritz-Hansen T, Sondergaard L, Jensen GB, Larsson HB (2001). Sustained postinfarction myocardial oedema in humans visualised by magnetic resonance imaging.. Heart.

[9] Dall’Armellina E, Karia N, Lindsay AC, Karamitsos TD, Ferreira V, Robson MD, Kellman P, Francis JM, Forfar C, Prendergast BD, Banning AP, Channon KM, Kharbanda RK, Neubauer S, Choudhury RP (2011). Dynamic changes of edema and late gadolinium enhancement after acute myocardial infarction and their relationship to functional recovery and salvage index.. Circ Cardiovasc Imaging.

[10] Zia MI, Ghugre NR, Connelly KA, Strauss BH, Sparkes JD, Dick AJ, Wright GA (2012). Characterizing myocardial edema and hemorrhage using quantitative T2 and T2* mapping at multiple time intervals post ST-segment elevation myocardial infarction.. Circ Cardiovasc Imaging.

[11] Payne AR, Casey M, McClure J, McGeoch R, Murphy A, Woodward R, Saul A, Bi X, Zuehlsdorff S, Oldroyd KG, Tzemos N, Berry C (2011). Bright-blood T2-weighted MRI has higher diagnostic accuracy than dark-blood short tau inversion recovery MRI for detection of acute myocardial infarction and for assessment of the ischemic area at risk and myocardial salvage.. Circ Cardiovasc Imaging.

[12] McAlindon EJ, Pufulete M, Harris JM, Lawton CB, Moon JC, Manghat N, Hamilton MC, Weale PJ, Bucciarelli-Ducci C (2015). Measurement of myocardium at risk with cardiovascular MR: comparison of techniques for edema imaging.. Radiology.

[13] Berry C Persistence of Infarct Zone T2 Hyperintensity at 6 Months After Acute ST-Elevation-Elevation Myocardial Infarction: Incidence, Pathophysiology and Prognostic Implications. 2017. American Heart Association. NCT02072850.. NCT02072850.

[14] Wassmuth R, Prothmann M, Utz W, Dieringer M, von Knobelsdorff-Brenkenhoff F, Greiser A, Schulz-Menger J (2013). Variability and homogeneity of cardiovascular magnetic resonance myocardial T2-mapping in volunteers compared to patients with edema.. J Cardiovasc Magn Reson.

[15] Dall’Armellina E, Piechnik SK, Ferreira VM, Si QL, Robson MD, Francis JM, Cuculi F, Kharbanda RK, Banning AP, Choudhury RP, Karamitsos TD, Neubauer S (2012). Cardiovascular magnetic resonance by non contrast T1-mapping allows assessment of severity of injury in acute myocardial infarction.. J Cardiovasc Magn Reson.

[16] Flett AS, Hasleton J, Cook C, Hausenloy D, Quarta G, Ariti C, Muthurangu V, Moon JC (2011). Evaluation of techniques for the quantification of myocardial scar of differing etiology using cardiac magnetic resonance.. JACC Cardiovasc Imaging.

[17] Francone M, Bucciarelli-Ducci C, Carbone I, Canali E, Scardala R, Calabrese FA, Sardella G, Mancone M, Catalano C, Fedele F, Passariello R, Bogaert J, Agati L (2009). Impact of primary coronary angioplasty delay on myocardial salvage, infarct size, and microvascular damage in patients with ST-segment elevation myocardial infarction: insight from cardiovascular magnetic resonance.. J Am Coll Cardiol.

[18] Payne AR, Berry C, Doolin O, McEntegart M, Petrie MC, Lindsay MM, Hood S, Carrick D, Tzemos N, Weale P, McComb C, Foster J, Ford I, Oldroyd KG (2012). Microvascular resistance predicts myocardial salvage and infarct characteristics in ST-elevation myocardial infarction.. J Am Heart Assoc.

[19] Carrick D, Haig C, Rauhalammi S, Ahmed N, Mordi I, McEntegart M, Petrie MC, Eteiba H, Lindsay M, Watkins S, Hood S, Davie A, Mahrous A, Sattar N, Welsh P, Tzemos N, Radjenovic A, Ford I, Oldroyd KG, Berry C (2015). Pathophysiology of LV remodeling in survivors of STEMI: inflammation, remote myocardium, and prognosis.. JACC Cardiovasc Imaging.

[20] García-Dorado D, Oliveras J, Gili J, Sanz E, Pérez-Villa F, Barrabés J, Carreras MJ, Solares J, Soler-Soler J (1993). Analysis of myocardial oedema by magnetic resonance imaging early after coronary artery occlusion with or without reperfusion.. Cardiovasc Res.

[21] Wisenberg G, Prato FS, Carroll SE, Turner KL, Marshall T (1988). Serial nuclear magnetic resonance imaging of acute myocardial infarction with and without reperfusion.. Am Heart J.

[22] Aletras AH, Tilak GS, Natanzon A, Hsu LY, Gonzalez FM, Hoyt RF, Arai AE (2006). Retrospective determination of the area at risk for reperfused acute myocardial infarction with T2-weighted cardiac magnetic resonance imaging: histopathological and displacement encoding with stimulated echoes (DENSE) functional validations.. Circulation.

[23] Bulluck H, Rosmini S, Abdel-Gadir A, White SK, Bhuva AN, Treibel TA, Fontana M, Ramlall M, Hamarneh A, Sirker A, Herrey AS, Manisty C, Yellon DM, Kellman P, Moon JC, Hausenloy DJ (2016). Residual myocardial iron following intramyocardial hemorrhage during the convalescent phase of reperfused ST-segment–elevation myocardial infarction and adverse left ventricular remodeling.. Circ Cardiovasc Imaging.

[24] Kali A, Kumar A, Cokic I, Tang RL, Tsaftaris SA, Friedrich MG, Dharmakumar R (2013). Chronic manifestation of postreperfusion intramyocardial hemorrhage as regional iron deposition: a cardiovascular magnetic resonance study with ex vivo validation.. Circ Cardiovasc Imaging.

[25] Kidambi A, Mather AN, Swoboda P, Motwani M, Fairbairn TA, Greenwood JP, Plein S (2013). Relationship between myocardial edema and regional myocardial function after reperfused acute myocardial infarction: an MR imaging study.. Radiology.

[26] Carrick D, Haig C, Ahmed N, Rauhalammi S, Clerfond G, Carberry J, Mordi I, McEntegart M, Petrie MC, Eteiba H, Hood S, Watkins S, Lindsay MM, Mahrous A, Welsh P, Sattar N, Ford I, Oldroyd KG, Radjenovic A, Berry C (2016). Temporal evolution of myocardial hemorrhage and edema in patients after acute ST-segment elevation myocardial infarction: pathophysiological insights and clinical implications.. J Am Heart Assoc.

[27] Carberry J, Carrick D, Haig C, Rauhalammi SM, Ahmed N, Mordi I, McEntegart M, Petrie MC, Eteiba H, Hood S, Watkins S, Lindsay M, Davie A, Mahrous A, Ford I, Sattar N, Welsh P, Radjenovic A, Oldroyd KG, Berry C (2016). Remote zone extracellular volume and left ventricular remodeling in survivors of ST-elevation myocardial infarction.. Hypertension.

[28] Fernández-Jiménez R, García-Prieto J, Sánchez-González J, Agüero J, López-Martín GJ, Galán-Arriola C, Molina-Iracheta A, Doohan R, Fuster V, Ibáñez B (2015). Pathophysiology underlying the bimodal edema phenomenon after myocardial ischemia/reperfusion.. J Am Coll Cardiol.

