# Supplementary material for: Persistence of Infarct Zone T2 Hyperintensity at 6 Months After Acute ST-Segment–Elevation Myocardial Infarction: Incidence, Pathophysiology, and Prognostic Implications
Source: Circ Cardiovasc Imaging. 2017 Dec 14;10(12):e006586. doi: 10.1161/CIRCIMAGING.117.006586 (PMC5753833; doi:10.1161/CIRCIMAGING.117.006586)
Supplement: Supplementary file 1 [file hci-10-e006586-s001.pdf]

SUPPLEMENTAL MATERIAL

Persistence of infarct zone T2 hyperintensity at 6 months after acute ST-elevation myocardial infarction: incidence, pathophysiology and prognostic implications

ClinicalTrials.gov registration NCT02072850

Table of contents

Supplementary Methods 3
Supplementary Results.. 11
Supplementary Figures 14
Supplementary Tables.. 18
References 26

Supplementary Methods

Setting and study populations

ST-elevation myocardial infarction patients

We performed a longitudinal cohort study in a regional cardiac center between 11 May 2011 and 22 November 2012. Patients with acute ST-elevation myocardial infarction (STEMI) were consecutively screened for suitability and those recruited provided written informed consent. Inclusion criteria were an indication for primary percutaneous coronary intervention (PCI) or thrombolysis for STEMI[1]. Exclusion criteria were contraindications to contrast-enhanced cardiac magnetic resonance (CMR) imaging. For the purposes of this analysis, STEMI patients who experienced a recurrent MI or had an additional PCI following the index procedure were not included since these events could influence myocardial T2 (ms) during the intervening period. STEMI management followed current guidelines[1,2]. The study was approved by the National Research Ethics Service (Reference 10-S0703-28) and was publically registered (NCT02072850). The flow diagram for the study is shown in Supplementary Figure 1.

Screening, enrolment, and data collection were prospectively performed by cardiologists in the cardiac catheterization laboratories of the Golden Jubilee National Hospital, Glasgow, United Kingdom. This hospital is a regional referral center for primary and rescue PCI. The hospital provides clinical services for a population of 2.2 million. A screening log was recorded, including patients who did not participate in the cohort study.

Coronary angiogram acquisition and analyses

Coronary angiograms were acquired during usual care with cardiac catheter laboratory X-ray (Innova®) and IT equipment (Centricity®) made by GE Healthcare.

Percutaneous coronary intervention

Consecutive admissions with acute STEMI referred for emergency PCI were screened for the inclusion and exclusion criteria. During ambulance transfer to the hospital, the patients received 300 mg of aspirin, 600 mg of clopidogrel and 5000 IU of unfractionated heparin[1,2]. The initial primary PCI procedure was performed using radial artery access. A conventional approach to primary PCI was adopted in line with usual care in our hospital[1,2]. Conventional bare metal and drug eluting stents were used in line with guideline recommendations and clinical judgment. The standard transcatheter approach for reperfusion involves minimal intervention with aspiration thrombectomy only or minimal balloon angioplasty (e.g. a compliant balloon sized according to the reference vessel diameter and inflated at 4-6 atmospheres 1-2 times). During PCI, glycoprotein IIbIIIa inhibitor therapy was initiated with high dose tirofiban (25 μg/kg/bolus) followed by an intravenous infusion of 0.15 μg/kg/min for 12 hours, according to clinical judgment and indications for bail-out therapy[1,2]. No reflow was treated according to contemporary standards of care with intra-coronary nitrate (i.e. 200 μg) and adenosine (i.e. 30 – 60 μg)[1,2], as clinically appropriate. In patients with multivessel coronary disease, multivessel PCI was not recommended, in line with clinical guidelines[1,2]. The subsequent management of these patients was symptom-guided.

Angiographic analysis

The coronary anatomy and disease characteristics of study participants were described based on the clinical reports of the attending cardiologist.

Outcome definitions

Coronary blood flow can be described based on the visual assessment of coronary blood flow revealed by contrast injection into the coronary arteries[1,2]. TIMI Coronary Flow Grade 0 is no

flow, 1 is minimal flow past obstruction, 2 is slow (but complete) filling and slow clearance, and 3 is normal flow and clearance.

CMR acquisition

CMR imaging was performed on a Siemens MAGNETOM Avanto (Erlangen, Germany) 1.5-Tesla scanner with a 12-element phased array cardiac surface coil.

Myocardial native longitudinal relaxation time (T1) reflects tissue water content and cellularity[3]. T1-mapping was performed pre- and 15 minutes post-gadolinium contrast administration. T1 maps were acquired in 3 short-axial slices (basal, mid and apical), using a modified look-locker inversion-recovery (MOLLI) investigational prototype sequence (Work-in-Progress (WIP) method 448, Siemens Healthcare)[4–6] that incorporates an automatic registration algorithm based on a previously described approach[7]. The MOLLI T1 cardiac-gated acquisition involved three inversion-recovery prepared look locker experiments combined within one protocol (3 (3) 3 (3) 5)[5]. The CMR parameters were: bandwidth ~1090 Hz/pixel; flip angle 35°; echo time (TE) 1.1 ms; T1 of first experiment 100 ms; TI increment 80 ms; matrix 192 x 124 pixels; spatial resolution 2.2 x 1.8 x 8.0 mm; slice thickness 8 mm; scan time 17 heartbeats.

Myocardial transverse relaxation time (T2) directly reflects tissue water content and mobility[8,9]. T2-mapping (WIP method 447, Siemens Healthcare) was acquired in contiguous short axis slices covering the whole ventricle, using an investigational prototype T2-prepared (T2P) TrueFisp sequence[8,9]. The CMR parameters were: bandwidth ~947 Hz/pixel; flip angle 70°; T2 preparations: 0 ms, 24 ms, and 55 ms respectively; matrix 160 x 105 pixels; spatial resolution 2.6 x 2.1 x 8.0 mm; slice thickness 8 mm.

Late gadolinium enhancement images covering the entire left ventricle (LV) were acquired 10-15 minutes after intravenous injection of 0.15 mmol/kg of gadoterate meglumine (Gd2+-

DOTA, Dotarem, Guebert S.A.) using segmented phase-sensitive inversion recovery (PSIR) turbo fast low-angle shot[10]. Typical imaging parameters were: bandwidth ~130 Hz/pixel, flip angle 25°, TE 3.36 ms, matrix 192 x 256 pixels, echo spacing 8.7ms and trigger pulse 2. The voxel size was 1.8 x 1.3 x 8 mm3. Inversion times were individually adjusted to optimize nulling of apparently normal myocardium (typical values, 200 to 300 ms).

CMR image analyses

The images were analysed on a Siemens work-station by observers with at least 3 years CMR experience (N.A., D.C., I.M, S.R.). All of the images were reviewed by experienced CMR cardiologists (C.B., N.T.). LV dimensions, volumes and ejection fraction were quantified using computer assisted planimetry (syngo MR®, Siemens Healthcare, Erlangen, Germany). All scan acquisitions were spatially co-registered.

ECV measurement

LV contours were delineated on the best spatially matched raw T1 image and copied onto color-coded spatially co-registered maps. Regions of interest were drawn in infarcted myocardium surrounding core, remote myocardium and LV blood pool. Hematocrit (HCT) was measured at the time of scanning. Extracellular volume (ECV) was calculated as a ratio of corresponding T1 values measured pre- and post- contrast in each of the regions of interest. ECV was calculated using ECV = (1-HCT) × λ, where Lambda (λ)=ΔR1myocardium/ΔR1blood, ΔR1=R1post-contrast-R1pre-contrast and R1=1/T1[11,12].

Infarct definition and size

The territory of infarction was delineated using a signal intensity threshold of >5 standard deviations (SD) above a remote reference region and expressed as a percentage of total LV mass[13]. Infarct regions with evidence of microvascular obstruction were included within the

infarct area and the area of microvascular obstruction was assessed separately and also expressed as a percentage of total LV mass.

Reference ranges

Reference ranges used in the laboratory were 105 – 215 g for LV mass in men, 70 – 170 g for LV mass in women, 77 – 195 ml for LV end-diastolic volume in men, 52 – 141 ml for LV end-diastolic volume in women, 19 – 72 ml for LV end-systolic volume in men and 13 – 51 ml for LV end-systolic volume in women.

Electrocardiogram

A 12 lead ECG was obtained before coronary reperfusion and 60 minutes afterwards with Mac-Lab® technology (GE Healthcare) in the catheter laboratory and a MAC 5500 HD recorder (GE Healthcare) in the Coronary Care Unit. The ECGs were acquired by trained cardiology staff. The ECGs were de-identified and transferred to the local ECG management system. The ECGs were then analysed by the University of Glasgow ECG Core Laboratory which is certified to ISO 9001: 2008 standards as a UKAS Accredited Organisation.

The extent of ST-segment resolution on the ECG assessed 60 minutes after reperfusion compared to the baseline ECG before reperfusion[1] was expressed as complete (≥70%), incomplete (>30% to < 70%) or none (≤30%).

Biochemical measurement of infarct size

Troponin T was measured (Elecsys Troponin T, Roche) as a biochemical measure of infarct size. The high sensitive assay reaches a level of detection of 5 pg/ml and achieves less than 10%variation at 14 pg/ml corresponding to the 99th percentile of a reference population. A blood sample was routinely obtained 12 – 24 hours after hospital admission, and again between 0700 - 0900 hours during the first two days of the index hospitalization.

Biochemical measurement of LV remodeling

Serial systemic blood sample were obtained immediately after reperfusion in the cardiac catheterization laboratory, and subsequently between 0600 - 0700 hrs each day during the initial in-patient stay in the Coronary Care Unit.

NT-proBNP, a biochemical measure of LV wall stress, was measured in a research laboratory using an electrochemiluminescence method (e411, Roche) and the manufacturers calibrators and quality control material. The limit of detection is 5 pg/ml. Long-term coefficient of variations of low and high controls are typically <5%, and were all within the manufacturers range.

Research Management

The study was conducted in line with Guidelines for Good Clinical Practice (GCP) in Clinical Trials[14].

Trial management included a Trial Management Group, and an independent Clinical Trials Unit. Day to day study activity was coordinated by the Trial Management Group who was responsible to the Sponsor which was responsible for overall governance and that the trial was conducted according to GCP standards.

Health outcomes

We prespecified adverse health outcomes that are pathophysiologically linked with STEMI. The primary composite outcome was major adverse cardiac events (MACE) defined as cardiac death, non-fatal myocardial infarction or heart failure hospitalization following the 6-month CMR scan. All-cause death or heart failure (heart failure hospitalization or defibrillator implantation) following the 6-month CMR scan was a secondary outcome.

Research staff screened for events from enrolment by checking the medical records and by contacting patients and their primary and secondary care physicians as appropriate. Each serious adverse event was reviewed by a cardiologist who was independent of the research team and blinded to all of the clinical and CMR data. The serious adverse events were defined according to standard guidelines[15].

Statistics

Continuous variables are described as mean±SD, if normally distributed, and median (Q1, Q3) otherwise. Categorical variables are described as n (%). Variables are described overall and by presence or absence of persistent T2 hyperintensity. Patient and angiographic characteristics and CMR findings were compared between groups with presence or absence of persistent T2 hyperintensity using independent sample t-tests or Mann-Whitney tests, as appropriate. Binary logistic regression was used to identify associates of persistent T2 hyperintensity. Multivariable linear regression analyses using the enter method were performed to identify associates of the change in infarct zone T2 and LV parameters. Linear regression assumptions were verified using standardized residual plots.

Random effects models were used to compute inter-rater reliability measures (inter-class correlation coefficient (ICC)) for the reliability of infarct zone T2 values measured independently by 2 observers in 20 randomly selected patients from the cohort.

Cox proportional hazards regression was used to explore potential associations between persisting T2 hyperintensity and health outcome. The proportional hazards assumption was verified using log-minus-log plots. For these plots, continuous variables were categorized as above and below the median.

All p-values were 2-sided. A p-value >0.05 indicated the absence of a statistically significant effect. The natural log was used in transformations of variables. Analyses were performed using SPSS version 22 for Windows (SPSS, Inc., Chicago, Illinois), or R v3.3.0.

Supplementary Results

CMR findings

The full list of CMR findings are summarized in Supplementary Table 1.

The association between change in LV ejection fraction and persisting T2 hyperintensity and the change in infarct zone T2 is shown in Supplementary Table 2.

Infarct zone T2 inter-observer reliability

Infarct zone T2 in a subgroup of 20 randomly chosen patients was independently measured by two observers. The intra-class correlation coefficient for reliability of infarct zone T2 was 0.92 (95% confidence interval (CI): 0.75, 0.97); $p<0.001$. Bland-Altman plots (Supplementary Figure 2) showed no evidence of bias. The coefficient of variation for infarct zone T2 was 7.4%.

Persistent T2 hyperintensity and extracellular volume

Infarct zone ECV was measured in 127 patients at baseline and 124 patients at follow-up (n=124 paired measurements). The characteristics of these patients were similar to the whole cohort (data not shown). Infarct zone ECV and T2 were associated at baseline (0.14 (0.04, 0.24); p=0.007) which likely reflects the early increase in infarct zone extracellular water content. Additionally, infarct zone ECV at 6 months was higher in patients with persisting T2 hyperintensity (Table 2). There was an association between the change in infarct zone ECV at 6 months compared to baseline and the change in T2 in the infarct zone (0.15 (0.05, 0.24); p=0.002; n=124). The directions of change in infarct zone T2 and infarct zone ECV were independent (Chi square; p=0.420). In the majority of patients, infarct zone T2 decreased

over time, which means those who had a smaller decrease in infarct zone T2 had a larger increase in infarct zone ECV.

Since ECV may also reflect extracellular collagen volume fraction[16], progressive extracellular fibrosis within the infarct zone may lead to an increase in ECV in the chronic phase post-STEMI. After adjustment for infarct zone ECV, infarct zone T2 was no longer a multivariable associate of LV remodeling. Accepting some loss of statistical power in this subset analysis, the potential explanations for this result may include 1) progressive infarct zone fibrosis is associated with persistent T2 hyperintensity at 6 months; 2) extracellular rather than intracellular edema is prominent in pathological remodeling post-MI and; 3) measurement error, since hematocrit, which is required to calculate ECV, may not be uniformly distributed in systemic blood and injured capillaries may allow varying amounts of formed blood elements to occupy the microvascular compartment.

Associates with adverse remodeling at 6 months

The clinical characteristics that were included in the multivariable model with adverse remodeling at 6 months were BMI (p=0.589), age (p=0.502), male sex (p=0.812), previous MI (p=0.708), previous PCI (p=0.347), diabetes mellitus (p=0.432), previous angina (p=0.879), hypertension (p=0.437), hypercholesterolemia (p=0.879), cigarette smoking (p=0.117), no ST-segment resolution vs. complete or partial ST-segment resolution (reference category) (p=0.882), TIMI coronary flow grade 0/1 pre-PCI vs. TIMI coronary flow grade 2/3 pre-PCI (reference category) (p=0.280), TIMI coronary flow grade 0/1/2 post-PCI vs. TIMI coronary flow grade 3 post-PCI (reference category) (p=0.295) systolic blood pressure at initial angiography per 10mmHg (p=0.419), heart rate (p=0.818), symptom onset to reperfusion time (p=0.258), percentage stenosis of culprit artery (p=0.274).

The multivariable predictors are described in Table 3 in the main paper.

Persistent T2 hyperintensity and health outcomes

Kaplan-Meier plots for the association between persisting T2 hyperintesity and all-cause death or heart failure and major adverse cardiac events are shown in Supplementary Figure 3.

Supplementary Figures

Supplementary Figure 1. CONSORT flow diagram.

Supplementary Figure 2. Bland-Altman plot for inter-observer variability in infarct zone T2 measurement.

Supplementary Figure 3. Kaplan-Meier plots for the association between persisting T2 hyperintesity and A) all-cause death or heart failure (Log rank = 0.115) and B) major adverse cardiac events (Log rank = 0.212).

Supplementary Figure 1.

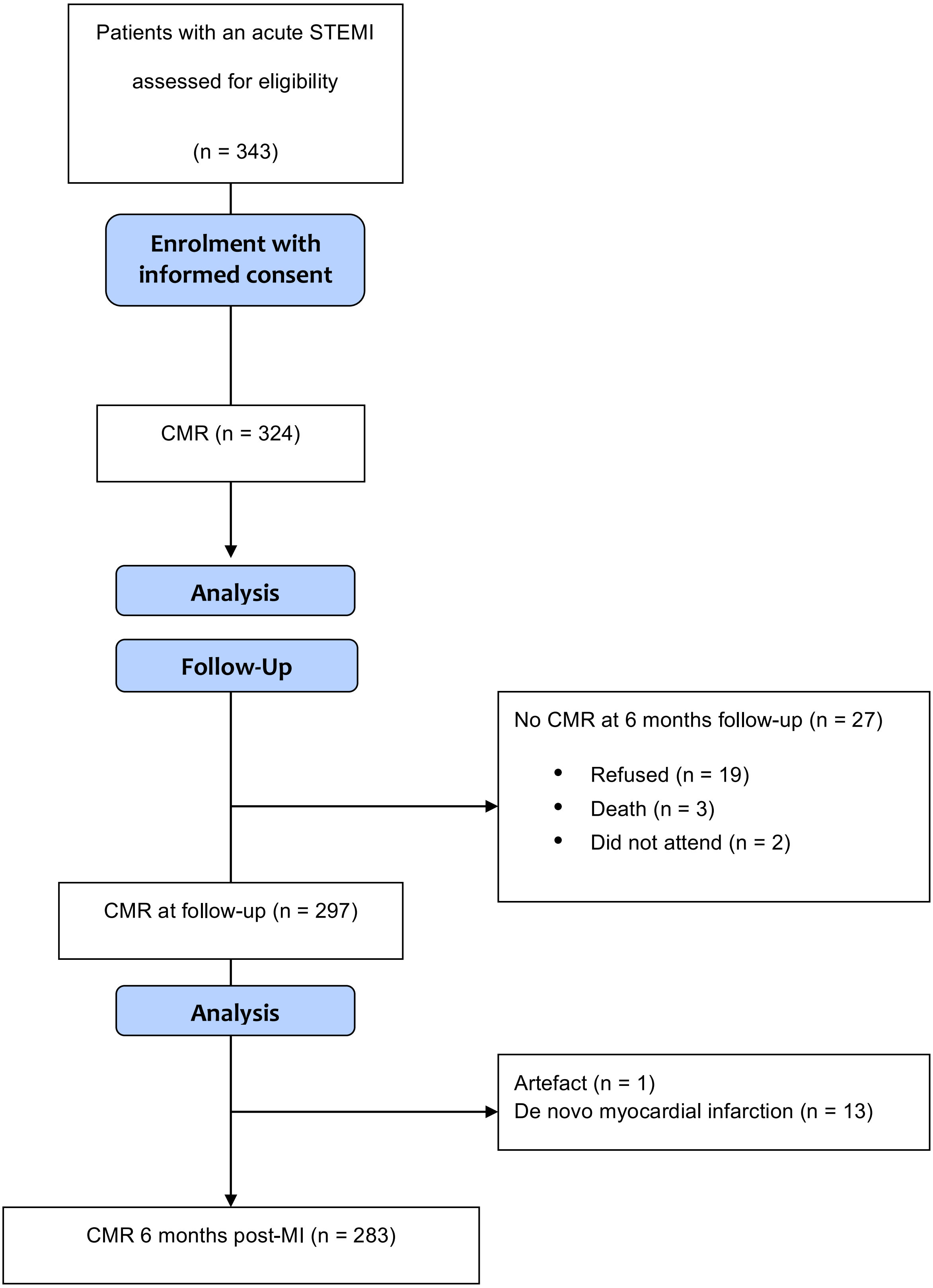

Supplementary Figure 2

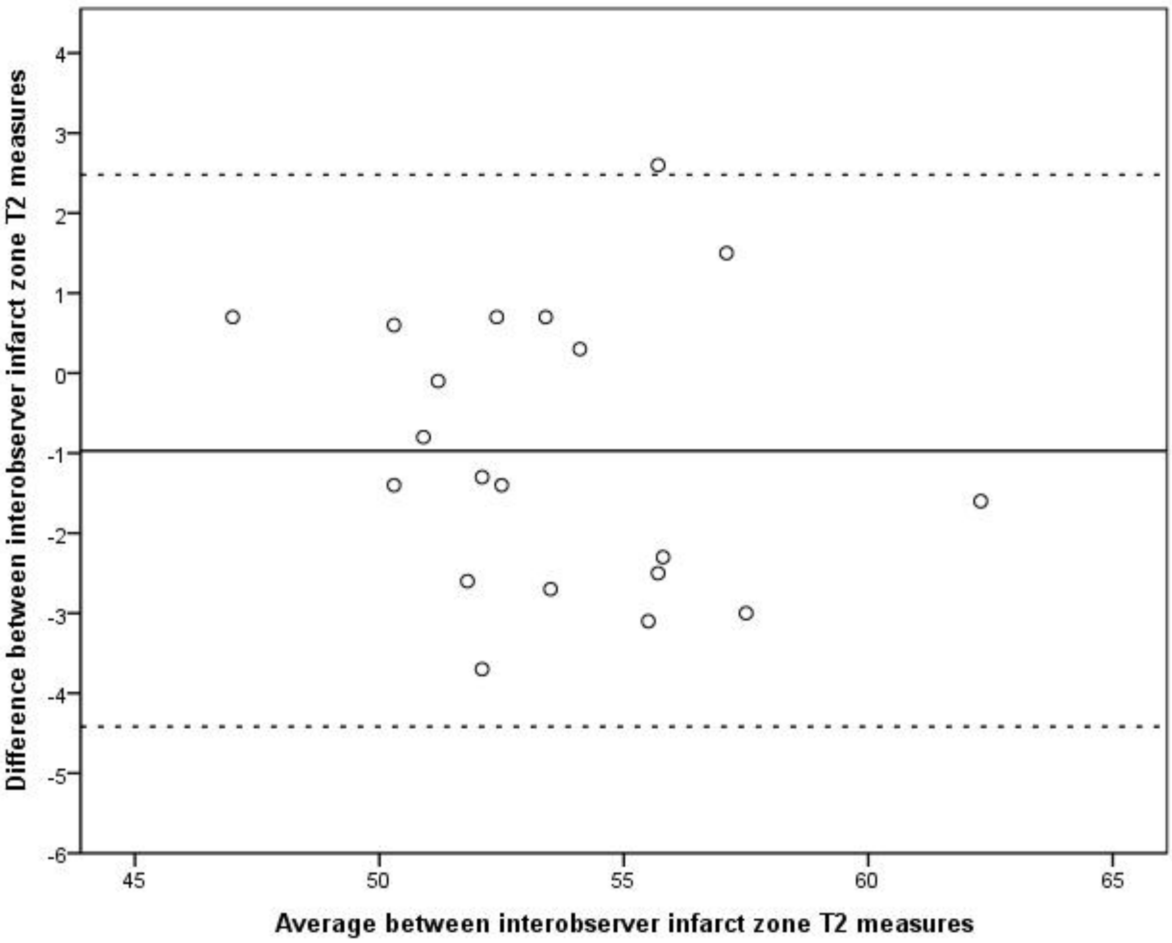

Supplementary Figure 3

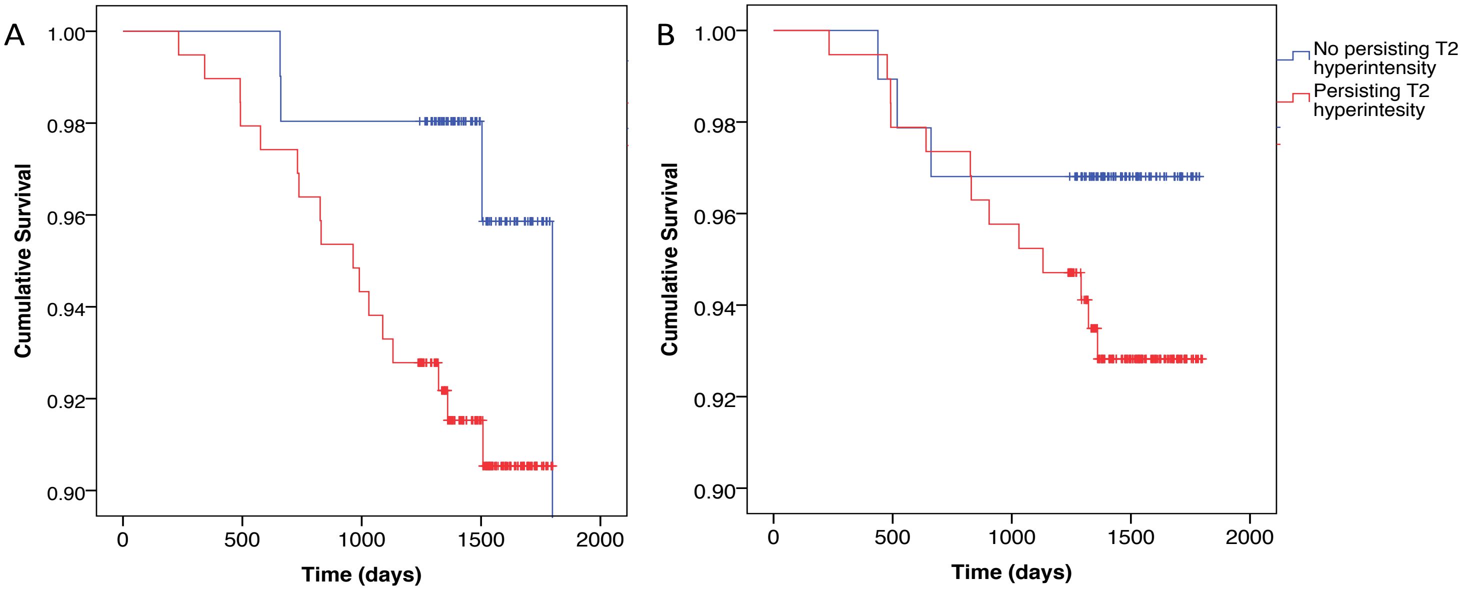

Supplementary Tables

Supplementary Table 1. CMR findings in 283 patients grouped according to the presence or absence of persistent T2 hyperintensity revealed by T2-mapping at 6 months post-STEMI.

Characteristics	All patients	No persistent T2 hyperintensity	Persistent T2 hyperintensity	P-value*
	n=283	n=94 (33%)	n=189 (67%)	
CMR findings 2 days post-MI				
LV ejection fraction, %	55±10	57±10	55±10	0.071
LV end-diastolic volume, ml				
Men	161±31	155±30	164±31	0.036
Women	124±25	130±23	121±25	0.172
LV end-systolic volume, ml				

Men	74±26	69±25	78±26	0.014
Women	54±18	57±18	52±17	0.225
LV mass, g				
Men	144±33	137±29	148±34	0.028
Women	97±21	101±17	95±23	0.216
Edema and infarct characteristics				
Myocardial edema, % LV mass	32±12	30±13	33±11	0.050
Infarct size, % LV mass	18±13	13±13	20±13	<0.001
Myocardial salvage, % LV mass	19±9	21±10	18±8	0.033
Myocardial salvage index, % LV mass	63±24	73±24	58±23	<0.001
Late microvascular obstruction present, n (%)	138 (49)	33 (35)	105 (56)	0.002

Late microvascular obstruction, % LV mass	2.6±4.6	1.5±3.6	3.1±4.9	0.004
Myocardial T1 and T2 values				
T1 remote, ms	960±26	958±25	961±26	0.354
T1 infarct, ms	1097±52	1093±49	1099±53	0.338
T1 hypointense core present, n (%)	137 (48)	33 (35)	104 (55)	0.002
T1 hypointense infarct core, ms	996±60	999±56	995±61	0.702
T2 remote, ms	49.7±2.1	49.8±2.2	49.7±2.1	0.811
T2 infarct, ms	66.3±6.1	64.4±5.7	67.3±6.1	<0.001
T2 hypointense core present, n (%)	165 (58)	41 (44)	124 (66)	0.001
T2 hypointense infarct core, ms	54.1±4.8	53.9±5.1	54.2±4.7	0.698
Myocardial ECV values at baseline				

ECV remote (all subjects), %	25.6±2.9	25.6±2.8	25.6±3.0	0.995
Men	25.2±2.9	25.0±2.7	25.3±2.9	0.612
Women	27.0±2.6	27.1±2.4	26.8±2.8	0.763
ECV infarct, %	56.0±11.7	52.8±12.9	57.7±10.7	0.024
ECV hypointense infarct core, %	43.2±12.6	44.8±15.1	42.5±11.7	0.537
CMR findings at 6 months				
LV ejection fraction at 6 months, %	62±9	65±8	61±10	<0.001
LV end-diastolic volume at 6 months, ml				
Men	169±42	151±31	177±45	<0.001
Women	127±30	125±22	128±34	0.627
LV end-systolic volume at 6 months, ml				

Men	68±35	54±19	74±38	<0.001
Women	46±18	45±18	47±18	0.550
Adverse remodeling, n (%)	32 (12)	1 (1)	31 (17)	<0.001
Infarct characteristics at 6 months				
Infarct size at 6 months, % LV mass	13±10	9±9	15±10	<0.001
Myocardial T1 and T2 values at 6 months				
T1 remote at 6 months, ms	957±29	957±28	958±29	0.713
T1 infarct at 6 months, ms	1058±66	1035±59	1068±67	<0.001
T2 remote at 6 months, ms	49.7±2.3	50.3±2.5	49.4±2.1	0.001
T2 infarct at 6 months, ms	56.8±4.5	53.5±3.4	58.5±4.0	<0.001
Myocardial ECV values at 6 months				

ECV remote at 6 months (all subjects), %	25.6±2.7	25.5±2.6	25.7±2.8	0.519
Men	25.3±2.7	25.0±2.6	25.4±2.7	0.288
Women	26.8±2.5	26.9±2.1	26.8±2.9	0.917
ECV infarct at 6 months, %	51.6±11.1	47.5±11.0	53.7±10.5	<0.001

Footnote: Abbreviations: CMR = cardiac magnetic resonance, ECV = extracellular volume, LV = left ventricle, T1 = longitudinal relaxation time, T2 = transverse relaxation time. Data are given as n (%) or mean±SD as appropriate. *P-values were obtained from two-sample t-test, Mann Whitney test or Fisher's test.

Supplementary Table 2. Linear regression analysis for associations with the change in LV ejection fraction at 6 months post-STEMI.

Multivariable associations	coefficient (95% CI)	p value
Patient characteristics, angiographic data and persistent T2 hyperintensity		
Persistent T2 hyperintensity	-2.53 (-4.39, -0.68)	0.008
Previous MI	6.29 (1.75, 10.83)	0.007
LV ejection fraction at baseline, %	-0.38 (-0.48, -0.29)	<0.001
Patient characteristics, angiographic data and change in infarct zone T2 (1 ms change)		
Change in infarct zone T2, 1 ms	-0.45 (-0.65, -0.26)	<0.001
Percentage stenosis of culprit artery, %	-0.14 (-0.24, -0.04)	0.007
Previous MI	5.47 (1.07, 9.86)	0.015
Hypertension	2.24 (0.31, 4.17)	0.023
Baseline infarct zone T2, ms	-0.52 (-0.73, -0.31)	<0.001
LV ejection fraction at baseline, %	-0.39 (-0.49, -0.30)	<0.001
Patient characteristics, angiographic data and change in infarct zone T2 (10 ms change)		
Change in infarct zone T2, 10 ms	-2.37 (-4.00, -0.75)	0.004
Percentage stenosis of culprit artery, %	-0.16 (-0.26, -0.05)	0.003
Previous MI	5.65 (1.14, 10.16)	0.014
Baseline infarct zone T2, ms	-0.36 (-0.55, -0.16)	<0.001
LV ejection fraction at baseline, %	-0.39 (-0.48, -0.29)	<0.001

Footnote: Abbreviations: CI = confidence intervals, LV = left ventricle, MI = myocardial infarction, T2 = transverse relaxation time. The coefficient (95% confidence intervals) indicates the magnitude and direction of the difference in change in LV ejection fraction (%) for the patient characteristic (binary or continuous).

The clinical characteristics that were included in the multivariable model with adverse remodeling at 6 months were BMI (p=0.574), age (p=0.354), male sex (p=0.460), previous PCI (p=0.808), diabetes mellitus (p=0.671), previous angina (p=0.402), hypertension (p=0.076), hypercholesterolemia (p=0.468), cigarette smoking (p=0.246), no ST-segment resolution vs. complete or partial ST-segment resolution (reference category) (p=0.746), TIMI coronary flow grade 0/1 pre-PCI vs. TIMI coronary flow grade 2/3 pre-PCI (reference category) (p=0.764), TIMI coronary flow grade 0/1/2 post-PCI vs. TIMI coronary flow grade 3 post-PCI (reference category) (p=0.529) systolic blood pressure at initial angiography per 10mmHg (p=0.578), heart rate (p=0.782), symptom onset to reperfusion time (p=0.617).

References

1. O'Gara PT, Kushner FG, Ascheim DD, Casey DE, Chung MK, Lemos JA de, Ettinger SM, Fang JC, Fesmire FM, Franklin BA, Granger CB, Krumholz HM, Linderbaum JA, Morrow DA, Newby LK, Ornato JP, Ou N, Radford MJ, Tamis-Holland JE, Tommaso CL, Tracy CM, Woo YJ, Zhao DX. 2013 ACCF/AHA Guideline for the Management of ST-Elevation Myocardial Infarction. *Circulation*. 2013;127:e362–e425.

2. King SB, Smith SC, Hirshfeld JW, Jacobs AK, Morrison DA, Williams DO, Members 2005 Writing Committee, Smith SC, Feldman TE, Hirshfeld JW, Jacobs AK, Kern MJ, King SB, Morrison DA, O'Neill WW, Schaff HV, Whitlow PL, Williams DO, Smith SC, Jacobs AK, Adams CD, Anderson JL, Buller CE, Creager MA, Ettinger SM, Halperin JL, Hunt SA, Krumholz HM, Kushner FG, Lytle BW, Nishimura R, Page RL, Riegel B, Tarkington LG, Yancy CW. 2007 Focused Update of the ACC/AHA/SCAI 2005 Guideline Update for Percutaneous Coronary Intervention. *Circulation*. 2008;117:261–295.

3. Moon JC, Messroghli DR, Kellman P, Piechnik SK, Robson MD, Ugander M, Gatehouse PD, Arai AE, Friedrich MG, Neubauer S, Schulz-Menger J, Schelbert EB. Myocardial T1 mapping and extracellular volume quantification: a Society for Cardiovascular Magnetic Resonance (SCMR) and CMR Working Group of the European Society of Cardiology consensus statement. *J Cardiovasc Magn Reson*. 2013;15:92.

4. Messroghli DR, Greiser A, Fröhlich M, Dietz R, Schulz-Menger J. Optimization and validation of a fully-integrated pulse sequence for modified look-locker inversion-recovery (MOLLI) T1 mapping of the heart. *J Magn Reson Imaging*. 2007;26:1081–1086.

5. Messroghli DR, Walters K, Plein S, Sparrow P, Friedrich MG, Ridgway JP, Sivananthan MU. Myocardial T1 mapping: Application to patients with acute and chronic myocardial infarction. *Magn Reson Med*. 2007;58:34–40.

6. Xue H, Guehring J, Srinivasan L, Zuehlsdorff S, Saddi K, Chefdhotel C, Hajnal JV, Rueckert D. Evaluation of rigid and non-rigid motion compensation of cardiac perfusion MRI. *Med Image Comput Comput-Assist Interv MICCAI Int Conf Med Image Comput Comput-Assist Interv*. 2008;11:35–43.

7. Chefd'hotel C, Hermosillo G, Faugeras O. Flows of diffeomorphisms for multimodal image registration. In: 2002 IEEE International Symposium on Biomedical Imaging, 2002. Proceedings. 2002. p. 753–756.

8. Giri S, Chung Y-C, Merchant A, Mihai G, Rajagopalan S, Raman SV, Simonetti OP. T2 quantification for improved detection of myocardial edema. *J Cardiovasc Magn Reson*. 2009;11:56.

9. Verhaert D, Thavendiranathan P, Giri S, Mihai G, Rajagopalan S, Simonetti OP, Raman SV. Direct T2 Quantification of Myocardial Edema in Acute Ischemic Injury. *JACC Cardiovasc Imaging*. 2011;4:269–278.

10. Kellman P, Arai AE, McVeigh ER, Aletras AH. Phase-sensitive inversion recovery for detecting myocardial infarction using gadolinium-delayed hyperenhancement†. *Magn Reson Med*. 2002;47:372–383.

11. Ugander M, Oki AJ, Hsu L-Y, Kellman P, Greiser A, Aletras AH, Sibley CT, Chen MY, Bandettini WP, Arai AE. Extracellular volume imaging by magnetic resonance imaging provides insights into overt and sub-clinical myocardial pathology. *Eur Heart J*. 2012;33:1268–1278.

12. Wong TC, Piehler K, Meier CG, Testa SM, Klock AM, Aneizi AA, Shakesprere J, Kellman P, Shroff SG, Schwartzman DS, Mulukutla SR, Simon MA, Schelbert EB. Association Between Extracellular Matrix Expansion Quantified by Cardiovascular Magnetic Resonance and Short-Term MortalityClinical Perspective. *Circulation*. 2012;126:1206–1216.

13. Flett AS, Hasleton J, Cook C, Hausenloy D, Quarta G, Ariti C, Muthurangu V, Moon JC. Evaluation of Techniques for the Quantification of Myocardial Scar of Differing Etiology Using Cardiac Magnetic Resonance. *JACC Cardiovasc Imaging*. 2011;4:150–156.

14. Medical Research Council. Guidelines for good clinical practice in clinical trials. [Internet]. [cited 2016 Jul 31];Available from: http://www.mrc.ac.uk/documents/pdf/good-clinical-practice-in-clinical-trials/

15. Hicks KA, Tcheng JE, Bozkurt B, Chaitman BR, Cutlip DE, Farb A, Fonarow GC, Jacobs JP, Jaff MR, Lichtman JH, Limacher MC, Mahaffey KW, Mehran R, Nissen SE, Smith EE, Targum SL. 2014 ACC/AHA Key Data Elements and Definitions for Cardiovascular Endpoint Events in Clinical Trials: A Report of the American College of Cardiology/American Heart Association Task Force on Clinical Data Standards (Writing Committee to Develop Cardiovascular Endpoints Data Standards). *J Am Coll Cardiol*. 2015;66:403–469.

16. Miller CA, Naish JH, Bishop P, Coutts G, Clark D, Zhao S, Ray SG, Yonan N, Williams SG, Flett AS, Moon JC, Greiser A, Parker GJM, Schmitt M. Comprehensive Validation of Cardiovascular Magnetic Resonance Techniques for the Assessment of Myocardial Extracellular Volume. *Circ Cardiovasc Imaging*. 2013;6:373–383.
